# Supplementary material for: Deletion of wfs1 Impairs Oligodendrocyte Precursor Cells Dorsal Distribution and Myelination Through the wfs1-hmgcs1 Axis in Zebrafish
Source: Biology (Basel). 2026 Aug 16;15(16):1402. doi: 10.3390/biology15161402 (PMC13510151; doi:10.3390/biology15161402)
Supplement: Supplementary file 1 [file biology-15-01402-s001.zip › File S1-Transcriptome sequencing data and differentially expressed genes (DEGs) in wfs1-- zebrafish at 5 dpf/2.KEGG/wfs1KOvsWT/DOWN/src/dre04520.html]

dre04520


- dre:569566

- Down regulated genes

569566(-1.00953785382545)

- dre:571431

- Down regulated genes

571431(-6.42862549702529)

- dre:245702

- Down regulated genes

245702\_1(-1.45433245048201)

- dre:436718

- Down regulated genes

436718\_1(-6.85571323128554)

- dre:436718

- Down regulated genes

436718\_1(-6.85571323128554)

- dre:792699

- Down regulated genes

792699(-1.84389282152821)

- dre:792699

- Down regulated genes

792699(-1.84389282152821)

- dre:793158

- Down regulated genes

793158(-6.94943122774273)

- dre:57934

- Down regulated genes

57934(-1.25539807424297)

- dre:57934

- Down regulated genes

57934(-1.25539807424297)

- dre:793158

- Down regulated genes

793158(-6.94943122774273)
- dre:792699

- Down regulated genes

792699(-1.84389282152821)

- dre:793158

- Down regulated genes

793158(-6.94943122774273)
- dre:792699

- Down regulated genes

792699(-1.84389282152821)

- dre:436718

- Down regulated genes

436718\_1(-6.85571323128554)

- dre:30429

- Down regulated genes

30429\_2(-1.78714384880949)

Close
